# From Single Cells to Diagnosis: Proteomics Technologies in the Multi‐Omics Landscape of Rare and Mitochondrial Diseases

**DOI:** 10.1002/jimd.70236

**Published:** 2026-08-24

**Authors:** Simon Wetzel, Sebastian Porsdam Mann, Florian A. Rosenberger

**Affiliations:** ^1^ Division of Molecular Metabolism, Department of Medical Biochemistry and Biophysics Karolinska Institutet Stockholm Sweden; ^2^ Science for Life Laboratory (SciLifeLab), Karolinska Institutet Stockholm Sweden; ^3^ Centre for Inherited Metabolic Diseases (CMMS) Karolinska University Hospital Stockholm Sweden; ^4^ Center for Advanced Studies in Bioscience Innovation Law (CeBIL) University of Copenhagen Copenhagen Denmark; ^5^ Center for Biomedical Ethics Yong Loo Lin School of Medicine, National University of Singapore Singapore Singapore

**Keywords:** diagnostics, mitochondria, omics ethics, rare diseases, single‐cell proteomics, spatial proteomics

## Abstract

Proteomics by mass spectrometry has rapidly matured from a niche method into a standard tool. The recent 10‐year trajectory of single‐cell proteomics has opened a new biological dimension for studying disease. Mitochondrial diseases, with their pronounced cell‐to‐cell heterogeneity, are particularly, well‐suited to these methods. Here, we discuss how this approach can serve as an orthogonal functional layer for rare disease diagnostics. We trace the evolution of rare disease diagnostics from biochemical enzyme assays through genomics, transcriptomics, proteomics and metabolomics, highlighting incremental gains in diagnostic yield from individual omics layers and their integration. We discuss the limitations of bulk approaches in capturing the functional consequences of genetic perturbations, the new opportunities opened up by single‐cell measurements and how spatial single‐cell proteomics can further enrich the biological signal of affected cells in diagnostic tissues. We observe that the persisting diagnostic gap reflects not only technological limitations but, increasingly, challenges in data sharing and infrastructure as well as interpretive frameworks for functional molecular evidence. In this context, we consider opportunities for artificial intelligence and the ethical dimensions of single‐cell proteomics in rare disease diagnostics. Finally, we propose a single‐cell deep visual proteomics (scDVP) framework for clinical diagnostics of rare diseases with cell‐to‐cell variability, arguing that mitochondrial diseases are an ideal proof‐of‐concept.

## Graphical abstract

Wetzel et al. outline how individual omics methods contribute to the diagnosis of patients with rare, and particularly mitochondrial diseases, with a focus on how spatial proteomics is joining this multi‐omics stack.

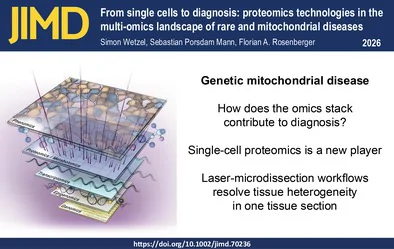

## 1 The Diagnostic Gap in the Rare Disease Field

Genome sequencing is now able to identify most variants in the human genome. However, whether a given variant explains a patient's phenotype is a different question entirely. Even after comprehensive genome investigation, 45%–65% of clinically suspected cases remain unsolved [1, 2, 3]. This gap persists despite rapid technological progress and is shaped by the realities of the rare disease field and limitations of clinical routines.

The field itself is dominated by the common among the uncommon, as approximately 80% of rare disease burden can be attributed to around 150 diseases [4]. The remaining diseases, estimated at more than 10 000 unique entries [5], are where diagnostic rates are likely lowest and where genotypically comparable patients may number only a handful worldwide. This causes a lack of well‐characterised reference cohorts, especially for patients that present with clinically atypical or multifactorial phenotypes. Standards for rare genetic disease diagnostics vary considerably across countries and healthcare systems. Whether a patient reaches the right national or local reference centre and whether new methods then reach patients depends on alignment with legal frameworks, ethical guidelines and cost‐effectiveness criteria. This translational process demands coordinated effort by large national and international initiatives [6, 7].

Understanding why a meaningful fraction of disease cases remains unsolved requires looking beyond pure yield statistics. Clinical sequence‐based pipelines are built on direct cause‐and‐effect frameworks which are robust for many genetic diagnoses. When reference data including clinical metadata does not exist at sufficient scale, these pipelines can frequently lead to uncertain genetic findings. Complex disease interactions present an intrinsic limitation that single variant‐centric frameworks cannot overcome. Also, an absent causative monogenic finding may not reflect technical failure but rather biological reality in the unknown fraction of undiagnosed patients. The downstream functional consequences of genetic variants, whether single, oligogenic or modulated by nuclear background and non‐genetic factors, can determine when a disease phenotype manifests or remains subclinical. The resulting disease complexity exceeds what current diagnostic pipelines are equipped to handle. The signal does not disappear but becomes indistinguishable from noise or it may depend on biological layers that genomic approaches do not fully capture.

Functional datasets can provide an orthogonal view to the gene layer, capturing pathway disturbances that allow us to disentangle interacting biological factors. The unresolved cases are where a functional layer and technological development are most required and where the diagnostic gap has the greatest clinical consequences. Rare diseases collectively affect 3%–6% of the population and, even conservatively estimated, an unresolved fraction of 20%–30% translates to more than 50 million individuals without a molecular diagnosis worldwide [4]. For these individuals, molecular diagnosis provides clinical value beyond curative treatment by informing disease management and genetic counselling [8, 9], while reducing the psychological burden of the diagnostic odyssey for patients and families [10, 11]. Moreover, molecular diagnosis provides the mechanistic foundation for therapeutic target discovery, underscoring why closing the diagnostic gap is central to progress in rare disease medicine.

## 2 Mitochondrial Disease as a Model for Rare Disease Diagnostic Complexity

Mitochondrial diseases are among the most common inborn errors of metabolism (IEMs) and represent a disease context in which these diagnostic challenges are particularly concentrated. Their prevalence is estimated at 23 cases per 100 000, with a minimal birth prevalence of at least 6.2 per 100 000 live births [12, 13]. The pathology most commonly arises from oxidative phosphorylation (OXPHOS) dysfunction, though the underlying disease mechanisms are diverse. These diseases can be caused by genetic variants in either mitochondrial DNA (mtDNA, adult prevalence of 20 in 100 000) or in nuclear DNA (nDNA, adult prevalence 2.9 in 100 000) genes required for mitochondrial function, maintenance or biogenesis [14]. The scale of this genetic complexity is reflected by MitoCarta 3.0 [15], a gene inventory which catalogues over 1100 human mitochondrial proteins, 13 of those mtDNA encoded. More than 350 nuclear genes are linked to human mitochondrial disease to date, though the true number is likely much higher [12].

The complexity of mitochondrial disease genetics is compounded by the mtDNA heteroplasmy phenomenon, which describes the coexistence of wild‐type and mutant mtDNA molecules within the same cell. In mtDNA disease, heteroplasmy levels vary across tissues, cell types and even over time, which interacts with disease burden in conjunction with mtDNA copy numbers [16, 17, 18]. In diagnostically relevant skeletal muscle biopsies, this presents functionally as a mosaic of OXPHOS‐deficient and histochemically normal fibres, which is a fundamental challenge for traditional one‐sample based diagnostic workflows.

## 3 A Matter of Methods

**FIGURE 1:**
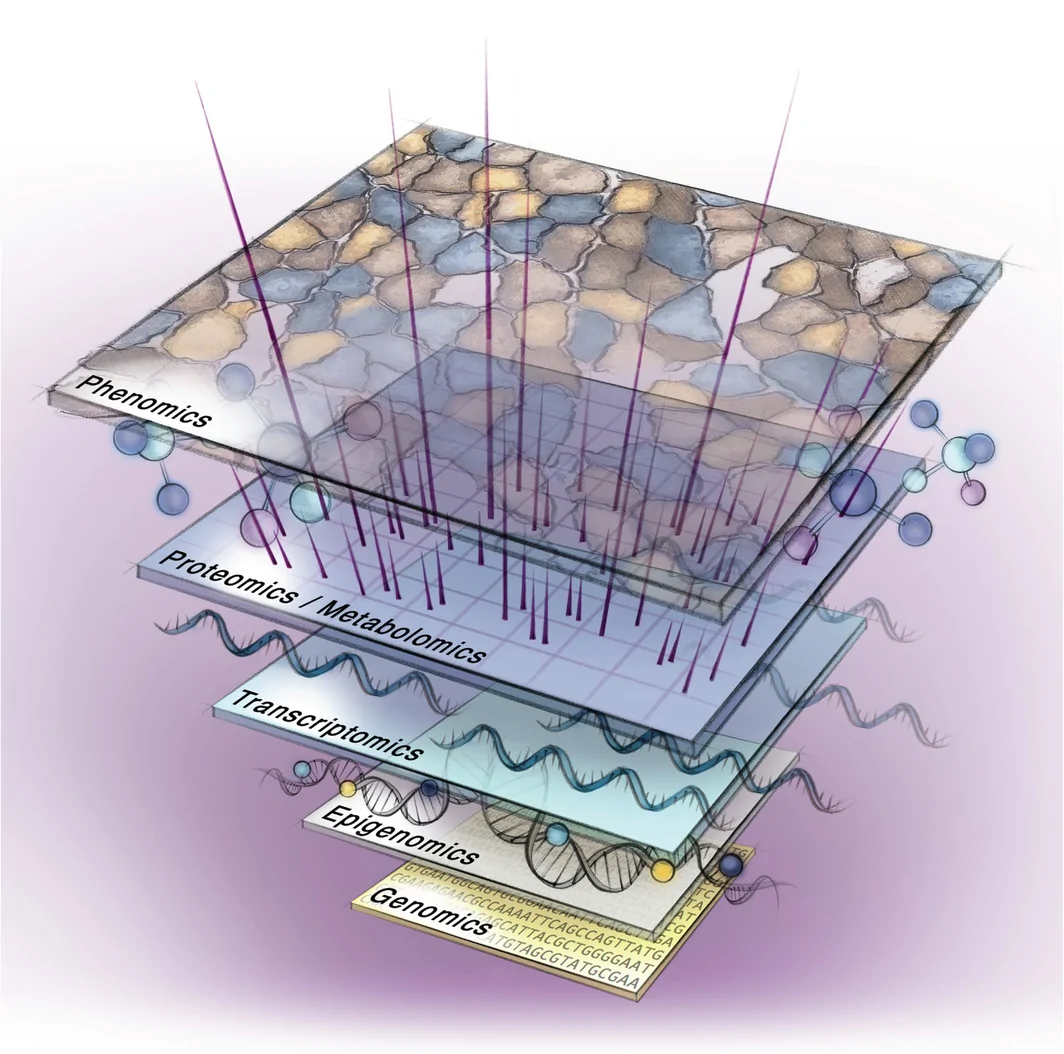
Multi‐omics layers in rare disease diagnostics. From genomic sequence to tissue phenotype. Omics data (genomics, epigenomics, transcriptomics, proteomics/metabolomics and phenomics) are ordered by increasing biological information and decreasing interpretability (from bottom to top).

Mass spectrometry‐based proteomics measures protein‐level consequences of genetic perturbations directly and in an unbiased manner, stacking on top of other omics layers (Figure 1). In parallel, single‐cell resolution can overcome the shortcomings of traditional approaches, where one static genetic snapshot is too rigid for a precise and mechanistic diagnosis. While mitochondrial diseases exemplify diagnostic challenges through genetic cell‐to‐cell heterogeneity, the diagnostic principles extend across most rare disease categories where tissue biopsies bring in an orthogonal functional layer. This review addresses the vision to use these functional omics measurements in combination with genetic diagnostics for increasing diagnostic yields of rare diseases through a technological lens.

### 3.1 The Pre‐Omics Diagnostic Toolkit

Rare disease diagnostics and, in particular, IEM diagnostics are built on a foundation of histochemistry, enzyme biochemistry and targeted sequencing methods. In mitochondrial diagnostics, histochemical staining of skeletal muscle biopsies, including cytochrome c oxidase (COX) and succinate dehydrogenase (SDH) staining assays, allows visualisation of OXPHOS dysfunction at the single fibre level [19]. In addition, biochemical measurements of respiratory chain enzyme activities in bulk tissue homogenates provide quantitative functional evidence of complex‐specific deficiencies [20]. For other IEMs, targeted enzyme activity measurements in blood, fibroblasts or leukocytes enable diagnosis of lysosomal storage diseases, glycogen storage disorders and fatty acid oxidation defects [21, 22, 23]. Direct functional measurement provides diagnostic information that sequence‐based approaches alone cannot provide. On the gene level, karyotyping and chromosomal microarray established the diagnostic framework in rare genetic diseases for detecting large structural variants and copy number changes. Chromosomal microarray remains today first‐tier testing for developmental delay and congenital anomalies, increasingly used alongside WES and WGS as part of a combined diagnostic approach [24].

### 3.2 The Sequencing Era in Rare Disease Diagnostics

Gene‐panel centric short‐read whole genome sequencing (srWGS) and whole exome sequencing (WES) is the current first‐line approach in broad clinical rare disease diagnostics. SrWGS offers a diagnostic yield advantage of 4%–10% over WES [1, 2, 3, 25], with cost‐effectiveness analyses favouring srWGS in several health system contexts due to shortened time to diagnosis [26]. State‐of‐the‐art srWGS gene panel approaches achieve a diagnostic yield of 30%–35% in heterogeneous study cohorts selected by high suspicion for rare disease [1, 3, 27]. However, this depends on phenotypic specificity, panel design and cohort composition: in mitochondrial diseases specifically, narrow gene panel approaches, restricted to known mitochondrial disease genes, achieve diagnostic yields of only 9%–16% [28, 29, 30]. This is largely because they cannot identify the 30%–60% of cases where non‐mitochondrial etiologies produce overlapping phenotypes [31, 32, 33]. Broader IEM gene panels or genome‐wide approaches reach diagnostic yields of 30%–37% in similar cohorts, capturing both mitochondrial and phenocopy diagnoses [31, 34, 35]. For rare diseases generally, routine clinical practice with gene‐panel centric WGS approaches achieves diagnostic yields of 20%–25% [36, 37], significantly lower than in most research studies. Contributing factors likely include broader referral criteria for genetic investigation and more heterogeneous cohort compositions, with clinical cohorts often encompassing a broader and more challenging disease spectrum.

Genome‐wide analysis broadens the search frame to unexpected genes but increases incidental finding burdens as well as interpretation time and computation costs, of which at least the first cannot be easily addressed in the AI era [38]. Trio analysis including genetic parents partially mitigates this by reducing variant interpretation time and improving yields by up to 9% compared to singleton approaches [39, 40, 41, 42], averaging around 40% in rare disease cohorts [43, 44]. Gene panels are recommended over genome‐wide srWGS as a first‐line approach in clinical phenotypes where both common non‐genetic and rare genetic causes overlap, such as in dilated cardiomyopathy [45]. This is in part because genome‐wide analysis carries a higher risk of incidental findings and can even lead to incorrectly assigned genetic diagnoses in phenotypically overlapping presentations. The guiding diagnostic principle is that false positive findings shall be avoided at all costs, because they can dramatically prolong the diagnostic journey and lead to wrong therapy. The incremental diagnostic yield of genome‐wide srWGS compared to optimised phenotype‐driven gene panels as a first‐line approach remains limited but understudied [39, 42, 46].

Recent studies report that genome‐wide reanalysis of initially negative WES or WGS yields 10%–25% additional diagnoses due to better algorithms, growing knowledge, technological maturation and improved international collaboration [47, 48, 49]. Of note, these figures derive from initially undiagnosed and then reanalysed cohorts, which is inherently confounded by temporal factors including new gene‐disease associations, improved bioinformatics and phenotypic evolution. The diagnostic rates of previously unsolved cases can be pushed higher through integration of reanalysed genomic sequencing with functional validation including model organisms, as shown by a study from the US‐based Undiagnosed Xiseases Network (UDN) achieving 35% [50]. Comparable rare disease diagnostic programmes exist internationally, including the overarching UDN international (UDNI) spanning many countries worldwide or the Solve‐RD consortium in Europe [51, 52]. These initiatives highlight the gap between existing work intensive, integrative and research‐grade approaches versus standard clinical diagnostic workflows.

### 3.3 Long‐Read Sequencing as Complementary Approach and srWGS Contender

SrWGS has inherent limitations in detecting complex structural variants and variants in repetitive or strongly enriched guanine and cytosine (GC‐extreme) regions and has limited interpretive capacity for deep‐intronic variants [53, 54, 55]. In contrast, the newer generation of long‐read WGS (lrWGS) addresses many of these blind spots, which now enables precise structural variant resolution, short tandem repeat (STR) sizing, phasing of compound heterozygotes and epigenetic profiling from a single assay [56, 57]. Clinical studies report that lrWGS identifies causative variants in 7%–18% of patients who remained undiagnosed after srWGS [58, 59, 60].

LrWGS is increasingly discussed as a potential successor to srWGS rather than a complementary tool, supported by recent advances in sequencing chemistry and instrumentation that have brought per‐base costs, sequencing depths and turnaround times towards srWGS parity [61, 62].

### 3.4 Transcriptome Sequencing in Rare Disease Diagnostics

RNA sequencing (RNA‐seq) detects three classes of findings that srWGS cannot directly measure: aberrant splicing, expression outliers reflecting haploinsufficiency or biallelic loss of function and monoallelic expression imbalance. Approximately 15% of disease‐causing variants are expected to affect mRNA splicing, with a substantial proportion outside canonical splice sites that are poorly predicted by current computational tools [63]. Diagnostic yield increases of 7%–17% have been reported in heterogeneous cohorts using blood or fibroblast transcriptomics [64, 65, 66, 67]. Sequencing of affected tissues itself almost doubled the diagnostic yield, yet remains constrained by tissue accessibility [68, 69]. Fibroblasts express around 60%–72% of Mendelian disease genes at sufficient depth [66, 67], exceeding 90% for mitochondrial disease genes [70], but in many rare neurological disorders causative genes are predominantly expressed in brain tissue [71]. In skeletal muscle, transcriptome sequencing has demonstrated particular diagnostic value; RNA‐seq of muscle biopsies identified the molecular diagnosis in approximately 35% of patients with suspected Mendelian muscle disease who remained undiagnosed after WES [72]. First formal guidelines for integrating RNA‐seq findings into the American College of Medical Genetics and Genomics (ACMG) and the Association for Molecular Pathology (AMP) variant classification have been proposed, supporting its gradual integration into clinical routine [73]. Deep learning‐based splice predictors, particularly, SpliceAI and autoencoder‐based methods for detecting aberrant splicing (e.g., FRASER) and expression outliers (e.g., OUTRIDER) are now essential tools for interpreting non‐coding variants and prioritising candidates for RNA‐seq validation [74, 75, 76].

**FIGURE 2:**
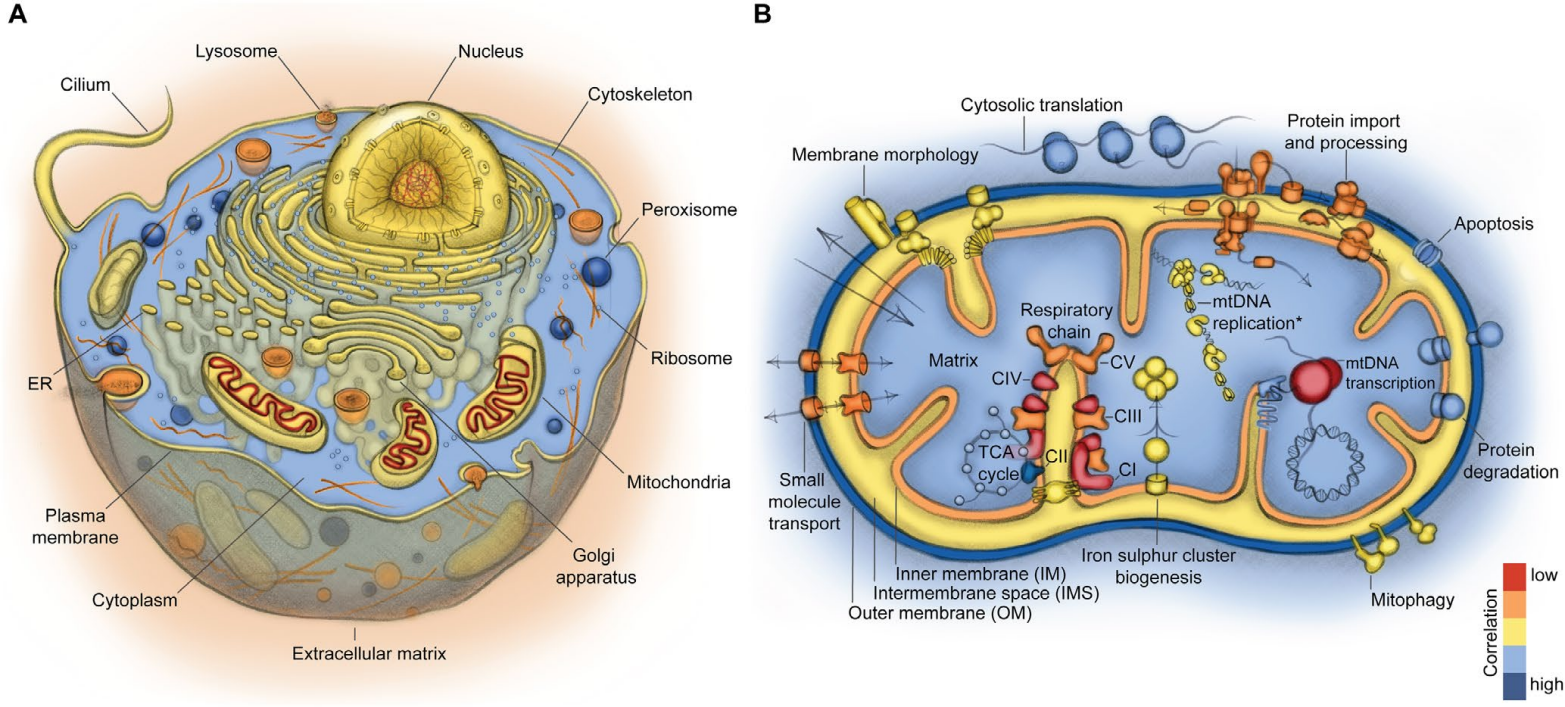
RNA‐protein correlation varies by subcellular compartment and is poor for OXPHOS complexes and the mitoribosome. (A) RNA‐protein correlation (Pearson's correlation coefficient *R* ^2^) across cellular organelles in mammalian cells and (B) across mitochondrial compartments. Correlation based on published transcriptomics and proteomics data from Ben‐Moshe et al. (A) [77] and Kühl et al. (A, B) [78]. (B) Design modified from Pfanner et al. (Figure 1) [79]. Five colour bins were used as indicated. *Correlation for mtDNA replication proteins inferred from mean mitochondrial protein correlation due to lack of representative amount of correlation data.

Quantitative RNA‐seq is often used as a predictor for protein levels, but its biological accuracy depends highly on the subcellular compartment of interest (median Pearson's *R* ^2^ ~ 0.26, Figure 2A) [77, 80, 81]. Particularly, in the mitochondrial disease group, RNA levels are very poor predictors of protein abundances in mitochondria, including OXPHOS and mitoribosomal proteins (Figure 2B) [78]. This highlights for mitochondrial disease diagnostics, in which OXPHOS dysfunction is a primary pathogenic mechanism, that transcriptomics alone cannot substitute for direct protein‐level measurement [77, 78].

### 3.5 Proteomics as Orthogonal Functional Evidence in Rare Disease

Quantitative MS‐based proteomics is transitioning from an analytical research field to a diagnostic contender with clinical relevance across multiple rare disease categories [82, 83]. MS‐based proteomics encompasses targeted approaches, which quantify predefined proteins with high sensitivity and reproducibility and untargeted approaches, which simultaneously profile thousands of proteins. MS‐based targeted approaches have seen limited adoption in rare disease proteomics, possibly constrained by the requirement for predefined biomarkers in very heterogeneous and difficult‐to‐classify referral cohorts. Affinity‐based proteomics platforms, including Olink Proximity Extension Assay and SomaScan [84, 85], offer a complementary and scalable targeted approach covering thousands of proteins from plasma or serum. Both platforms have been applied in rare disease from clinical trial to research settings, identifying candidate biomarkers across conditions including hereditary transthyretin amyloidosis, spinal muscular atrophy and Duchenne muscular dystrophy [86, 87, 88]. Their reliance on predefined affinity reagent panels and requirement for soluble input material restricts their use to body fluids, excluding spatially defined tissue analysis and unbiased profiling of intracellular disease mechanisms. Untargeted proteomics partially addresses these constraints by identifying protein abundance patterns that generate functional hypotheses—independently of prior genomic findings. Two distinct performance metrics are relevant: analytical sensitivity in molecularly confirmed disease, which establishes whether a disease‐specific proteomic signature exists and diagnostic yield in genomically unsolved cases to establish clinical utility.

Proof‐of‐concept exists across several rare disease contexts. In lysosomal storage disorders (LSDs), where proteo‐lipidomic profiling has characterised autophagy defects and secondary metabolic changes across diverse LSD mutations [89]. Deep plasma proteomics in Fabry disease has revealed systemic and tissue‐specific protein signatures using targeted profiling of over 1400 proteins [90]. Neuromuscular diseases have been studied using serum and tissue proteomics, with large‐scale studies in Duchenne muscular dystrophy identifying novel biomarkers that correlate with disease progression [88]. The translational potential of untargeted proteomics has been recently illustrated by a large clinical study in dystonia patients, in which MS‐based fibroblast proteomics led to higher diagnostic yields through both candidate variant reclassification and untargeted outlier analysis in cases missed by genomic approaches alone [91].

IEMs represent a conceptually fitting area for diagnostic proteomics integration, with mitochondrial diseases among the most extensively studied. Untargeted MS‐based proteomics of peripheral blood mononuclear cells demonstrated functional proteomic evidence in 83% of patients with molecularly confirmed mitochondrial disease. The authors identified putative biomarkers and disease‐group‐specific signatures, even when the causative protein itself was undetectable [92]. Fibroblast proteomics can provide proteomic classification by biochemical and genetic subtype and variant of uncertain significance (VUS) reclassification, following the ACMG/AMP variant classification framework in mitochondrial disease [93, 94]. Alongside RNA‐seq, fibroblast proteomics contributed to molecular diagnosis in 21% of 121 individuals in an undiagnosed heterogeneous cohort of WES‐negative Mendelian disease cases [95]. These proteomic signals are detectable regardless of whether the underlying genetic cause has been identified, making proteomics informative both in unsolved cases and in patients carrying VUS.

Despite emerging research‐grade discoveries, translation of proteomic signatures into routine clinical diagnostics remains an open challenge across both affinity‐based and MS‐based approaches, with clinical implementation achieved in a small number of specific disease contexts [86, 96, 97, 98]. Selected specialised diagnostic centres have begun deploying quantitative proteomics in specific cases, particularly where genomic findings are inconclusive and functional evidence is required [99]. The ACMG/AMP framework includes specific codes (PS3/BS3) for functional evidence and recent consensus recommendations have established calibration methods for quantifying the strength of functional assay data in variant interpretation [100]. Proteomic signatures of OXPHOS complex deficiency, mitochondrial import defects or pathway disruption can provide supportive functional evidence that contributes to variant pathogenicity assessment, though integration of proteomics into standardised ACMG/AMP frameworks remains an area of active development.

### 3.6 Metabolomics as Direct Readout of Biological Dysfunction

Metabolomics is the only omics layer that directly measures metabolic dysfunction as a chemical phenotype, without inferring consequences from upstream molecular changes. In IEM diagnostics, targeted metabolite quantification is already clinically established. Newborn screening programmes based on tandem MS detect inborn conditions from dried blood spots at population scale and targeted metabolite panels remain first‐line diagnostic tools for organic acidurias, fatty acid oxidation disorders and amino acid metabolism defects [101, 102].

Proof‐of‐concept studies established that a single, untargeted plasma MS assay could identify characteristic signatures across a broad IEM spectrum [103] and untargeted approaches have now demonstrated diagnostic value across approximately 100 distinct IEMs in research settings [104]. In multi‐omics integration frameworks, metabolomic profiles provide a direct readout of pathway dysfunction that, in the future, will complete the canonical omics descriptor. Integration frameworks combining targeted and untargeted metabolomics with genomic data have demonstrated high diagnostic accuracy in IEM cohorts and established methodological templates for metabolomics‐genomics VUS integration [105].

Technical boundaries currently constrain the clinical utility of untargeted approaches. The Human Metabolome Database catalogues over 220 000 unique compounds [106] and human plasma is estimated to contain over 4600 distinct metabolites, of which current single‐platform pipelines robustly identify around 1000 [104, 107]. Most detected signals cannot be confidently assigned to a known metabolite at the moment [104, 108] and tissue metabolome coverage from diagnostic biopsies is lower than plasma figures suggest [109].

Despite these prospects, metabolomics is currently capped by intrinsic shortcomings, such as rapid fluctuations of metabolite levels, complex setups for measuring flux instead of steady‐state and the inherent spectral complexity. Overcoming these will allow untargeted metabolomics to reach its full diagnostic potential. Importantly for this review, the protein and metabolite layers are tightly interconnected, with proteomics capturing the acting machinery and metabolomics reporting on its functional output.

## 4 When One Biological Layer Is Not Enough

No single omics layer captures the full complexity of rare disease biology. Genomics identifies candidate variants but cannot measure their functional consequence. Transcriptomics captures gene expression but only incompletely measures protein abundance or activity. Proteomics measures the functional protein landscape but does not resolve metabolic steady‐state or flux. Each layer is therefore informative but inherently incomplete. Integration of multiple layers has demonstrated additive diagnostic yield in prospective cohorts. In heterogeneous rare disease populations, adding functional omics to genome sequencing has increased diagnostic yields by approximately 7% [99], though gains vary substantially with cohort selection, tissue availability and the deployed omics layers.

The prevailing integration strategy remains a sequential filtering approach where genomic findings anchor interpretation and functional omics layers provide supporting or contradicting evidence [110]. Principled frameworks for weighing conflicting signals across layers remain underdeveloped and prospective clinical validation of multi‐omics pipelines is currently limited [99]. The sequential multi‐omics approach retains genomics as the interpretive gatekeeper, limiting the diagnostic contribution of functional layers to cases where a genomic candidate already exists. This excludes precisely the unsolved cases where functional evidence is most needed. Platforms such as MatchMaker Exchange have begun addressing this for phenotype‐to‐gene matching [111], enabling clinicians to query whether other centres have encountered patients with variants in the same gene and similar clinical presentations. Extending this model to functional omics data would address a fundamental challenge in rare disease proteomics: distinguishing true disease signatures from patient‐specific variation, biological and technical noise and secondary compensatory responses. A parallel bottleneck lies in structured clinical phenotyping, including systematic use of ontology‐based tools such as the Human Phenotype Ontology [112], without which multi‐omics patterns cannot be adequately anchored in clinical rare disease diagnostics [113].

## 5 From Bulk Quantification to Spatial Resolution

The utility of bulk quantitative proteomics is bounded by averaging signal across thousands of cells and multiple cell types, obscuring the spatial and cellular heterogeneity that defines disease at the tissue level. Particularly, in mitochondrial disease caused by variants in mtDNA, heteroplasmic phenotypes are then obscured. Similarly, we lose cell–cell interactions, paracrine signalling between diseased and healthy cells and microenvironmental influences on protein expression when tissue architecture is homogenised [114, 115].

Single‐cell resolution addresses these limitations by providing information fundamentally inaccessible to bulk measurements. Resolving protein abundance at the single‐cell level enriches the biological signal from phenotypically affected cells only. This inherently increases the statistical power of single‐cell over bulk approaches. In addition, measuring multiple cellular states from a single biopsy allows us to reconstruct temporal disease trajectories of spatially segregated cell states. This approach exemplifies mapping disease progression across spatial gradients within a single biopsy [114, 115, 116, 117].

Antibody‐based immunofluorescence approaches, including quadruple staining for OXPHOS complexes in skeletal muscle, have demonstrated the diagnostic value of tissue resolved protein tracking in muscle biopsies [118], a paradigm now extended to unbiased MS‐based proteomics. Advances in liquid chromatography‐mass spectrometry (LC–MS)‐based proteomics and laser microdissection technologies with high autonomy in cell selection have expanded the applicability of untargeted proteomics to low‐input samples, while preserving their spatial tissue context [114, 115, 116, 119, 120]. Deep visual proteomics (DVP), an LC–MS‐based approach, combines morphology‐guided single‐cell isolation with high‐sensitivity data‐independent acquisition MS (DIA‐MS) [114, 116, 117]. DVP enables unbiased, deep proteome profiling of defined cells when at least one stratifying marker protein is known. Over the past years, DIA‐MS has become the standard for data acquisition due to its systematic quantification of all detectable precursor ions within defined mass windows. DVP is operational down to the single cell level. Our work on hepatocytes could identify up to 4300 proteins per cell (median about 2700) from a 10 μm slice of a cell [121]. These capabilities position DVP, particularly its single‐cell branch, as a candidate diagnostic method for conditions with prominent cell‐to‐cell heterogeneity or cell‐type specific pathogenicity.

## 6 Spatial Single‐Cell Proteomics in Mitochondrial Disease as a Proof‐Of‐Concept

**FIGURE 3:**
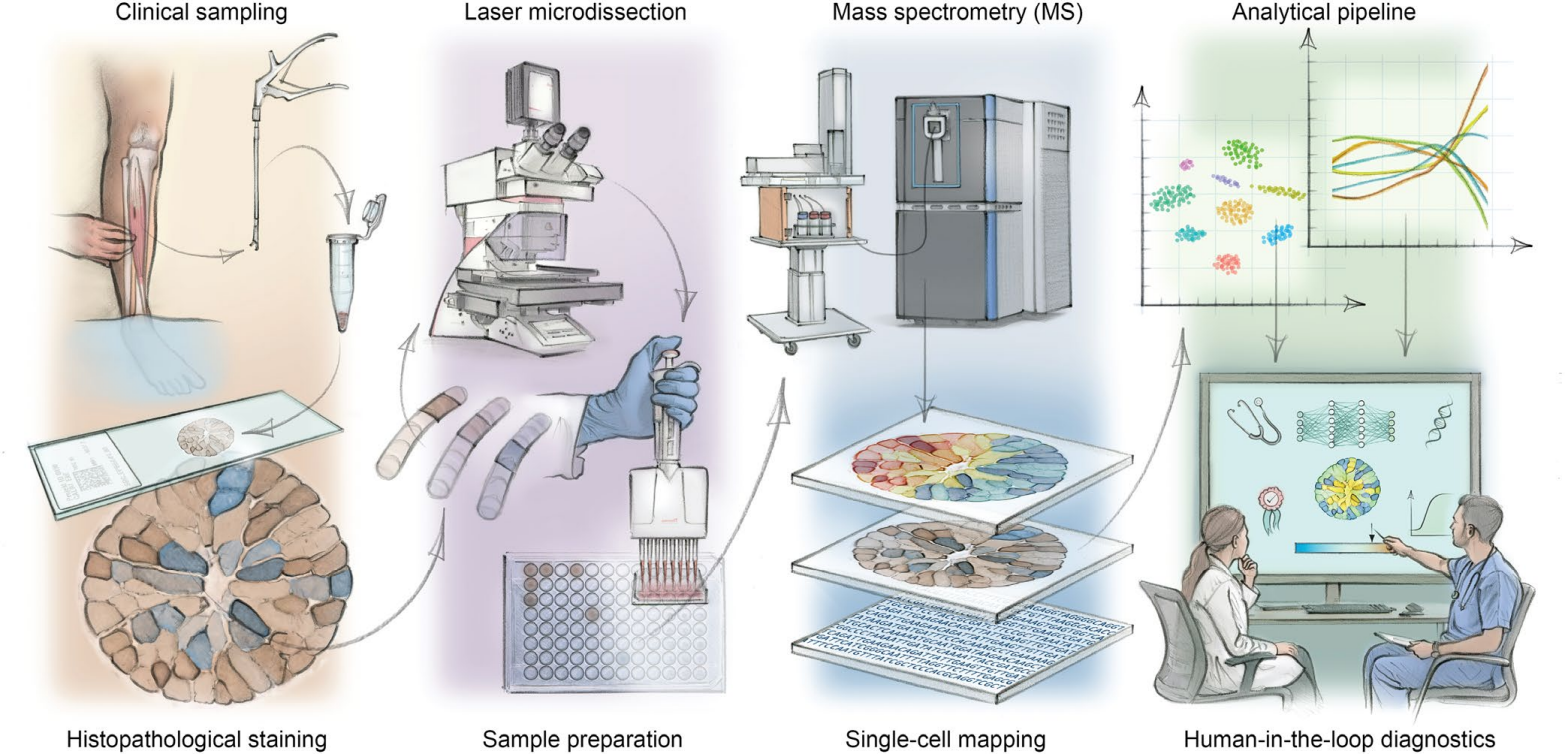
Spatial single‐cell proteomics workflow in mitochondrial disease diagnostics as proof‐of‐concept. Tissue acquisition from human skeletal muscle (m. tibialis anterior) and sample preparation of histological cross‐sections. Guided by histopathological staining, laser microdissection isolates single fibres that are collected into 384‐well plates for automated sample preparation. Following protein digestion, peptide samples are analysed by high‐sensitivity LC–MS, generating quantitative proteomic profiles. Single‐cell proteomic data are visualised as a layered heatmap reflecting protein abundances across the tissue section and underlying genomics profile. Automated protein pattern recognition, patient clustering and variant reprioritisation.

We propose a diagnostic single‐cell DVP (scDVP) framework for mitochondrial disease using skeletal muscle biopsies that remain the single, most informative diagnostic material [12, 122, 123, 124] (Figure 3). First, we use standard histochemical staining of fresh‐frozen muscle cross‐sections to assess respiratory chain activity at the level of individual fibres. Staining intensity is used to stratify fibres along a continuum from histochemically normal to severely deficient cells, defining single fibres for subsequent fully automated laser microdissection. Proteomic data is then acquired by DIA with downstream label‐free protein‐level quantification. Fibre protein profiles are ordered along the imaging‐based mitochondrial dysfunction trajectory, generating a pseudotime representation of mitochondrial decompensation within a patient's single biopsy. This allows the tracking of pathway‐level proteomic changes including OXPHOS subunit loss, assembly factor depletion and compensatory pathway upregulation from early, subtle stages to advanced fibre dysfunction. This approach extracts disease landmarks from cross‐sectional biopsies without requiring longitudinal sampling.

The newly obtained functional layer can now be used for variant reprioritisation and mechanistic hypothesis generation in genomically unsolved cases. The final assessment remains with medical expert teams (human‐in‐the‐loop diagnostics). It is now to be tested whether this output reaches diagnostic‐grade sensitivity and specificity.

The now publicly available DVP dataset in the Human Proteome Atlas demonstrates an average mitochondrial proteome coverage of 70% across 27 cell types in 14 tissues, with skeletal muscle specifically showing higher coverages for OXPHOS subunit proteins and mitochondrially encoded proteins [125]. For scDVP applied to single fibres, mitochondrial proteome coverage has not yet been systematically established. Coverage is expected to be lower than cell‐type resolved DVP given the lower input material, though our proof‐of‐concept data suggest that meaningful mitochondrial proteome depth (> 35%) is achievable at the single‐fibre level.

## 7 From Proof‐of‐Concept to Clinical Translation

Targeted MS‐based tissue proteomics has reached clinical implementation in, for instance, amyloidosis [86], where laser microdissected regions‐of‐interest serve disease subtyping. DVP extends this principle to unbiased, proteome‐wide profiling at single‐cell resolution. In skin, DVP applied to archived biopsies from toxic epidermal necrolysis (TEN) patients resolved the proteome of keratinocytes and infiltrating immune cells separately, identifying JAK/STAT and interferon signalling as primary pathogenic drivers. This informed JAK inhibitor treatment and was associated with recovery in at least seven TEN patients [115]. In liver, DVP applied to alpha‐1 antitrypsin deficiency (AATD) biopsies resolved molecular events across hepatocyte stress stages, identifying early peroxisomal activation as a candidate protective mechanism against fibrosis progression and demonstrating that protein accumulation is largely cell‐intrinsic [117]. These findings illustrate how spatial proteomics can identify actionable pathogenic mechanisms and stratify patients by disease trajectory within a single biopsy. Cancer, though not typically classified as a rare disease, exemplifies the principle: tumour heterogeneity at the cellular and regional level drives therapeutic resistance and cannot be captured by bulk profiling in diagnostic tissues. DVP applied to melanoma [114], colorectal cancer [126] and pancreatic cancer precursor lesions [127] has defined disease progression, immune evasion mechanisms and molecular transitions between cell states.

The same principles extend to tissues where current diagnostic approaches are limited by cellular heterogeneity or insufficient functional resolution. Beyond mitochondrial disease, skeletal muscle biopsies are guideline‐recommended for subtype classification of inflammatory myopathies. Even with solid clinical pre‐test probability, muscle biopsies fail to confirm an idiopathic inflammatory myopathy (IIM) subtype in around one third of cases [128]. In cardiac sarcoidosis, the endomyocardial biopsy limits histological diagnosis to 25%–38% of all examined cases [129]. Cell‐type‐level proteomic resolution could complement classification where histology is negative. More broadly, any tissue context combining guideline‐recommended biopsy indications with diagnostic limitations attributable to cellular heterogeneity represents a candidate application for single‐cell resolved proteomic analysis.

Practical barriers to clinical implementation include the specialised instrumentation required, estimated at €2 million for a complete DVP workflow (laser microdissection system, nanoflow LC, high‐resolution mass spectrometer and sample preparation automation). We hypothesise that by analysing up to 50 single cells per biopsy, DVP provides enough statistical power to resolve disease trajectories and distinguish pathological from normal cell populations. Current throughput of up to 840 single cells per week (LC‐limited) for deep proteome profiling allows 15–20 biopsies per week per LC–MS setup [130]. Given the historic development of LC–MS technology, it is likely we will see at least two doublings of throughput with identical or even higher protein depth over the next 5 years. Additionally, shorter LC gradients with less deep proteomes might be equally informative for diagnostic applications. Data storage and bioinformatics processing represent another infrastructure consideration. At 15–20 biopsies per week per LC–MS setup, raw Orbitrap Astral files generate more than 1 TB weekly, requiring GDPR‐compliant, access‐controlled infrastructure analogous to frameworks already established for clinical sequencing data. Similar to other omics or imaging data, suitable data formats and compression methods without sacrificing valuable diagnostic information are needed for large‐scale clinical translation [131]. Spectral library search and label‐free quantification of, for example, a 50‐fibre biopsy batch requires several hours on standard multi‐core workstation hardware, where processing time scales with library complexity and available computational resources [132]. Cloud‐deployable frameworks and emerging search engines demonstrate faster local execution with equivalent quantitative performance and are reducing computational infrastructure barriers for smaller diagnostic centres [132, 133, 134].

Several semi‐automated tools are now available that reduce manual intervention across DIA data processing, quality control and reporting steps [135, 136, 137, 138]. For DVP in particular, ongoing efforts aim to increase pipeline autonomy and accessibility by integrated open‐access software following FAIR principles (Findable, Accessible, Interoperable and Reusable) [139]. To harness the full diagnostic potential of untargeted proteomics datasets, adaptive frameworks for growing clinical cohorts have been proposed for biomarker reclassification using per‐sample feature selection and cohort cross‐validation [140]. While a fully integrated end‐to‐end pipeline from raw file to interpretable clinical output does not yet exist for single‐cell DVP in rare disease diagnostics, these tools collectively provide a credible path towards diagnostic‐grade automation.

## 8 AI‐Assisted Interpretation and Its Boundaries

AI technologies will fundamentally transform the interpretation of high‐dimensional biological datasets. Single‐cell proteomic data is inherently multivariate, simultaneously measuring thousands of proteins across spatially defined cell populations. Machine learning (ML) approaches are positioned to exploit this through identification of co‐abundance patterns, spatial gradients and cell‐type‐specific signatures that conventional statistical analysis cannot capture. Further downstream, omics‐driven disease classification requires annotated databases of sufficient scale and diversity before it can be meaningfully deployed, encompassing reference atlas‐based diagnosis, outlier flagging, candidate prioritisation and integration of discordant evidence across omics layers. Realising this potential requires two parallel foundations to mature: the technological workflows to generate single‐cell proteomic data at diagnostic scale and the data and reference infrastructure to clinically interpret it.

Despite its potential, ML approaches that learn directly from clinically annotated cases cannot easily extend beyond the knowledge those cases encode. The cases that remain unsolved are precisely those for which no such annotated signal exists, as they have not been diagnosed at sufficient scale or biological detail to generate learnable patterns. The diagnostic gap is therefore defined by what falls outside existing knowledge.

Emerging sequence‐to‐function models such as AlphaGenome extend this boundary by operating on a different type of ground truth [141]. Rather than learning from clinical databases, they predict molecular consequences from experimental measurements of gene expression, chromatin accessibility and splicing across hundreds of cell types. This allows functional hypotheses to be generated for diagnostically uncharacterised variants, including deep intronic and regulatory variants. Such tools can guide diagnostics in complex unresolved cases where sufficient functional evidence is currently lacking.

The underlying computation of AI prediction tools involves millions of weighted parameters. Explainability methods can identify which input features most influenced a prediction, but feature importance cannot be directly mapped to a biological mechanism [142]. AI‐generated reasoning that is statistically plausible but lacking a verifiable mechanism does not meet clinical diagnostic standards. When such outputs are incorrect, the absence of a traceable mechanism makes the error difficult to identify and correct.

This creates a notable double standard, as human diagnostic reasoning also relies on pattern recognition and clinical intuition that often cannot be mechanistically traced [143, 144]. Human diagnostic errors are estimated at 8%–15% depending on specialty and setting [145, 146, 147], with inter‐physician diagnostic variability well‐documented across medical fields [148, 149, 150]. Recent studies show that AI systems can exhibit cognitive biases similar to those seen in human clinicians, sometimes even more pronounced [151]. Thus, the focus should be on developing AI systems with error pattern recognition but not demand complete mechanistic transparency that human reasoning itself cannot provide.

## 9 Ethical Dimension

Ethical guidance in clinical proteomics has focused on the handling of sensitive and potentially identifiable data, the representativeness of reference databases and equitable access to diagnostic benefits [152, 153, 154, 155, 156]. While these are relevant here, three features distinguish single‐cell proteomics in rare disease from this broader setting.

First, tissue acquisition is guided by a specific diagnostic question rather than broadly sampled, which tends to narrow the surface on which incidental findings arise without eliminating it. Second, the patient population is radically smaller: in ultra‐rare disease, comparable patients may number only a handful worldwide. Third, the diagnostic signal is functional rather than genotypic, which reshapes how uncertainty in the data should be reported and acted on. These shifts interact, bringing three issues into focus: incidental findings in deep functional profiles, the near n‐of‐one character of rare disease diagnostics and findings of uncertain significance.

Incidental findings arise in deep functional profiling by design rather than by accident. In broader work on clinical proteomics, the principal axis for deciding how such findings should be returned has been the distinction between actionable and unactionable findings. In a tissue‐biopsy context, a second axis becomes salient alongside it, which we might call the distinction between near‐indication and far‐indication findings. A muscle biopsy for suspected mitochondrial disease may reveal an inflammatory signature close to the clinical question or a signal bearing on a remote condition. Findings of the first kind arguably fall within the scope of the original consent; findings of the second kind often do not. In practice, a ‘need to know’ principle often overrides this where findings are both actionable and of serious health consequence. In rare disease diagnostics, the index case is frequently a child and untargeted methods generate too wide a range of possible findings for any single consent encounter to anticipate. Consequently, decisions about late‐onset findings and the right not to know in practice often fall to expert teams weighing in individual patient risks based on disease‐specific and ethical guidelines. A child's prospective autonomy, a patient's best interests and the interests of other family members for whom an actionable finding may be relevant are all factors to account for when considering the degree and kind of return of information.

In biomedical research, privacy is typically treated as a primary ethical constraint and rare disease diagnostics, with their near n‐of‐one cohorts, concentrate the problem. A published phenotype combined with a deep proteomic profile will often identify a patient without any explicit identifier, since anonymisation depends on a crowd that does not exist at this scale. Standard privacy frameworks are not well positioned to resolve this tension. Designed for population‐scale data, they treat identifiability as a property to be reduced; in n‐of‐one settings, the information density that enables diagnosis is also what enables identification. International sharing of functional omics data is the most effective lever available for closing the diagnostic gap, since each new diagnosis often depends on a small number of prior profiles. The third issue concerns findings of uncertain significance, which arise here in two distinguishable forms. The first is a familiar inheritance from clinical genetics, a variant for which the evidence of pathogenicity is incomplete. Functional proteomic data, by providing a direct readout of the pathway consequences of such a variant, should help to resolve a proportion of these cases as evidence accumulates. The second form is newer and more specific to proteomic profiling itself: patterns at the protein or pathway level that are statistically robust, but whose mechanistic basis cannot be recovered from the data. A useful analogy is the incidentaloma in clinical imaging, where one accepted response to a finding of unclear significance is to report it as such and direct it to follow‐up rather than force an interpretation the underlying data cannot support.

These three issues each turn on the same underlying point. Guidelines that are either unduly restrictive or insufficiently specified to be predictable foreclose the studies that would close the diagnostic gap they are meant to protect, at a cost paid disproportionately by patients already poorly served. Responsible self‐regulation, informed by patient and advocate perspectives, offers the field a route to rules it can stand behind. The moment to do this work is while the methods and their clinical indications are still being defined, rather than after they have settled into a shape that is harder to revise.

## 10 A Call for Functional Evidence

The diagnostic gap in rare genetic disease will not be closed by genome sequencing alone. Improving variant detection and interpretation has driven diagnostic yield gains substantially. The remaining unsolved cases could now profit from functional evidence that genomic data inherently cannot provide. The field has responded by developing orthogonal molecular approaches, each measuring a different biological layer and the evidence supports that integrating additional data layers increases diagnostic yield beyond what any single method achieves [66, 67, 92, 95, 99, 157].

Proteomics resolves dysfunction at the level of proteins, protein complexes and pathways, situated between transcriptomic and metabolomic evidence. Spatial single‐cell proteomics solves the shortcomings of bulk proteomics by resolving functional consequences at the cellular tissue level.

Mitochondrial diseases serve us as a proof of concept, where genetic complexity, nuclear‐mitochondrial interactions and spatially heterogeneous tissue dysfunction create a diagnostic challenge that illustrates both the limitations of sequencing alone and the complementary value of functional evidence.

Rapid technological advances have shifted the proteomics field's limiting factor from analytical depth towards biological interpretation and clinical integration. On the technology side, advances in spatial isolation, low‐input sample preparation and throughput are converging into workflows such as DVP, enabling the multivariate pattern recognition that high‐dimensional spatial proteomic data demands. On the data infrastructure side, tissue proteome atlases, disease cohort profiling, cross‐ancestry reference databases and standardised reporting must be built before reference‐based profiling and multi‐omics disease classification become tractable. Each molecularly resolved rare disease case contributes to building the databases. Generating data at scale will require not only infrastructure investment and clinical validation but coordinated data‐sharing frameworks across international rare disease cohorts. The work of building this infrastructure is at once scientific and ethical, determining not only what novel methods can do but also who will benefit from them and how reliably they will do so.

## Author Contributions

S.W. and F.A.R. conceived the review and the figures. S.P.M. drafted the ethics section. S.W. drafted the manuscript under the guidance of F.A.R. All authors contributed to revision and approved the final version of the manuscript.

## Funding

This work was supported by Research and Life Science Council of Region Stockholm (FoUI‐1025541), Swedish Research Council (2024‐02522), Swedish Society for Medical Research (SG‐24‐0048‐B‐H‐01), Novo Nordisk Foundation (NNF25OC0105216) and Karolinska Institutet.

## Conflicts of Interest

The authors declare no conflicts of interest.

## Data Availability

Data sharing not applicable to this article as no datasets were generated or analysed during the current study.
